# Where Vision Meets Memory: An Eye-Tracking Study of In-App Ads in Mobile Sports Games with Mixed Visual-Quantitative Analytics

**DOI:** 10.3390/jemr18060074

**Published:** 2025-12-10

**Authors:** Ümit Can Büyükakgül, Arif Yüce, Hakan Katırcı

**Affiliations:** Department of Sports Management, Eskisehir Technical University, Eskişehir 26555, Turkey; uc_buyukakgul@eskisehir.edu.tr (Ü.C.B.); hakankatirci@eskisehir.edu.tr (H.K.)

**Keywords:** eye-tracking, in-app advertising, mobile sports games

## Abstract

Mobile games have become one of the fastest-growing segments of the digital economy, and in-app advertisements represent a major source of revenue while shaping consumer attention and memory processes. This study examined the relationship between visual attention and brand recall of in-app advertisements in a mobile sports game using mobile eye-tracking technology. A total of 79 participants (47 male, 32 female; Mage = 25.8) actively played a mobile sports game for ten minutes while their eye movements were recorded with Tobii Pro Glasses 2. Areas of interest (AOIs) were defined for embedded advertisements, and fixation-related measures were analyzed. Brand recall was assessed through unaided, verbal-aided, and visual-aided measures, followed by demographic comparisons based on gender, mobile sports game experience and interest in tennis. Results from Generalized Linear Mixed Models (GLMMs) revealed that brand placement was the strongest predictor of recall (*p* < 0.001), overriding raw fixation duration. Specifically, brands integrated into task-relevant zones (e.g., the central net area) achieved significantly higher recall odds compared to peripheral ads, regardless of marginal variations in dwell time. While eye movement metrics varied by gender and interest, the multivariate model confirmed that in active gameplay, task-integration drives memory encoding more effectively than passive visual salience. These findings suggest that active gameplay imposes unique cognitive demands, altering how attention and memory interact. The study contributes both theoretically by extending advertising research into ecologically valid gaming contexts and practically by informing strategies for optimizing mobile in-app advertising.

## 1. Introduction

Technological advancements have not only provided new tools for understanding consumer behavior but have also enabled the development of digital platforms that have become an integral part of everyday life [1]. With the widespread use of smartphones and mobile internet, mobile applications and games have emerged as core components of modern digital culture [2,3]. Today, individuals rely on mobile applications for a wide range of activities such as socializing, obtaining information, shopping, banking, and following global trends [4,5,6,7,8]. Offering fast, personalized, and interactive experiences, mobile applications foster stronger user engagement compared to other digital systems [9].

Among mobile applications, mobile games represent one of the fastest-growing segments of the digital economy. With an annual growth rate of 3%, the global mobile gaming market reached USD 92.6 billion in 2024, accounting for approximately 48% of total global gaming revenues (including PC, console, and mobile platforms) [10]. According to Statista Market Insights [11], this figure is projected to exceed USD 126 billion by the end of 2025.

The growing number of individuals downloading and actively playing mobile games has led to a diversification of mobile marketing practices and the emergence of new advertising strategies [12,13]. Within this context, mobile advertisements—specifically in-app advertising integrated into the flow of mobile applications—have become one of the most influential tools of digital marketing [14]. The Interactive Advertising Bureau defines in-app advertising as “ads and ad campaigns that are delivered within mobile applications, including smartphones, tablets, or wearable devices” [15]. These advertisements allow users to access information about promoted products, services, or brands while engaging with the app itself. In-app advertisements can thus be defined as ad messages delivered seamlessly within the natural flow of a mobile application, without interrupting user interaction [16]. As visually integrated elements, such ads are capable of attracting attention dynamically, producing measurable engagement outcomes, and establishing stronger user–brand interaction [17,18].

Previous research indicates that in-app advertising is among the most frequently used monetization strategies in mobile games [19,20,21,22,23]. Furthermore, such advertisements have been found to positively influence purchase intentions, brand recommendation behaviors, and brand rankings [9,24]. They also enhance users’ emotional experiences, fostering more favorable attitudes toward brands [9]. Collectively, these factors underscore the relevance of investigating in-app advertising from a cognitive and perceptual perspective—particularly regarding its influence on brand recall [18,25,26,27,28].

Within the mobile gaming ecosystem, sports games hold a distinctive position due to their interactive and immersive nature [29]. For developers, such games represent a vital source of ad revenue, while for consumers, advertising effectiveness largely depends on brand recall. Brand recall refers to an individual’s ability to spontaneously (unaided) or prompted (aided) remember a brand [30]. In the marketing and advertising literature, recall is regarded as the first step in a consumer’s brand evaluation and decision-making process [31,32,33]. However, in attention-demanding and cognitively intensive contexts such as mobile sports games, advertising success depends not only on exposure frequency but also on the degree to which the advertisement captures and sustains user attention [34].

Most prior studies on brand recall have focused on traditional media such as television or desktop environments [35,36,37]. Yet mobile games represent a distinct context characterized by fragmented attention, rapid decision-making, and high cognitive load. In this setting, eye-tracking emerges as a powerful method, providing objective metrics such as time to first fixation, fixation duration, and fixation count. Beyond measuring where and how long individuals look, visualization of eye movement data provides a critical analytical layer that helps interpret cognitive and perceptual mechanisms. Visualization techniques such as heatmaps, scanpath plots, and gaze trajectory diagrams translate raw gaze coordinates into meaningful spatial–temporal representations, revealing attention distribution and information-processing dynamics that would otherwise remain invisible. Recent advances in gaze visualization have enhanced the interpretability of eye-tracking findings across diverse domains, including advertising, gaming, and human–computer interaction [38,39,40,41]. These approaches not only support quantitative evaluation but also allow researchers to qualitatively examine how users allocate visual attention within complex, interactive environments [42,43]. Although eye-tracking has been widely used in consumer and advertising research, studies directly linking visual attention to brand recall in mobile sports gaming contexts remain scarce. Moreover, most existing research has examined passive viewing (e.g., watching e-sports), while neglecting the active playing experience, which involves different attentional and cognitive mechanisms.

Building upon these quantitative gaze measures, visualization offers a complementary interpretive layer that bridges raw gaze data and cognitive understanding. It reveals spatial–temporal attention patterns that numeric summaries alone cannot capture [44]. Recent work further emphasizes attention-aware visualization, where evolving user perception informs analysis and design decisions in real time [45]. In this study, grounded in perceptual and cognitive principles [46] and visual analytics foundations [47], we integrate heatmaps (attentional density) and gaze plots/scanpaths (temporal sequencing) to explore how visual attention relates to brand recall in active gameplay.

Therefore, this study examines the relationship between visual attention and brand recall in embedded in-app advertisements during active gameplay in mobile sports games. Employing mobile eye-tracking in a naturalistic play context enhances ecological validity and provides insight into the interplay between attention, cognition, and memory in digital environments.

Crucially, this study advances beyond standard descriptive eye-tracking by employing a mixed visual–quantitative analytic framework. While previous HCI research has often treated fixation metrics and visual scans as separate entities, we integrate Generalized Linear Mixed Models (GLMMs) with spatial visualization to model the predictive validity of gaze behavior. This approach offers a specific contribution to vision research by quantifying how ‘active vision’ in dynamic tasks differs from passive viewing, specifically testing whether fixation duration remains a reliable predictor of memory when attentional resources are constrained by gameplay mechanics.

The study specifically addresses the following research questions:

- ***RQ1*:** Does brand recall (unaided/aided) differ across demographic or behavioral variables (gender, mobile sports game experience and interest in tennis)?
- ***RQ2*:** How do individual differences (e.g., gender, tennis interest) relate to visual attention metrics, and to what extent do gaze-based measures (TFD, TTFF) predict visual-aided brand recall when controlling for brand placement?

## 2. Materials and Method

### 2.1. Experimental Design

The primary aim of this study was to examine the recall of in-app advertisements embedded in mobile sports games. The study examined whether brand recall for in-app advertisements embedded in a mobile sports game differed by gender, mobile sports game experience (MSGE), and interest in tennis. It was hypothesized that the cognitive load inherent in active gameplay would generate distinct attentional and memory outcomes compared to passive viewing contexts.

### 2.2. Participants

A total of 79 participants (47 males and 32 females; Mage = 25.8, SD = 5.5) took part in the experiment, recruited via convenience sampling. Inclusion criteria required normal or corrected-to-normal vision and no ocular impairments that would prevent wearing eye-tracking glasses. Additionally, participants were required to have basic familiarity with smartphone and tablet use. Before the experiment, they were informed about the duration of the session, the mobile sports game to be played, and that their eye movements would be recorded while playing. Ethical approval for this study was obtained from the Health Sciences Institute Ethics Committee of (corresponding author institution anonymized for blind review) (Approval No: 68215917-050.99). All participants were informed about the study’s general purpose, procedures, and confidentiality measures, and provided written informed consent prior to participation, in accordance with the Declaration of Helsinki. To ensure data privacy, raw eye-tracking video recordings containing identifiable facial features are stored on a secure, password-protected server accessible only to the research team, while only de-identified numerical gaze data are used for public sharing.

### 2.3. Experimental Stimuli

**Figure 1:**
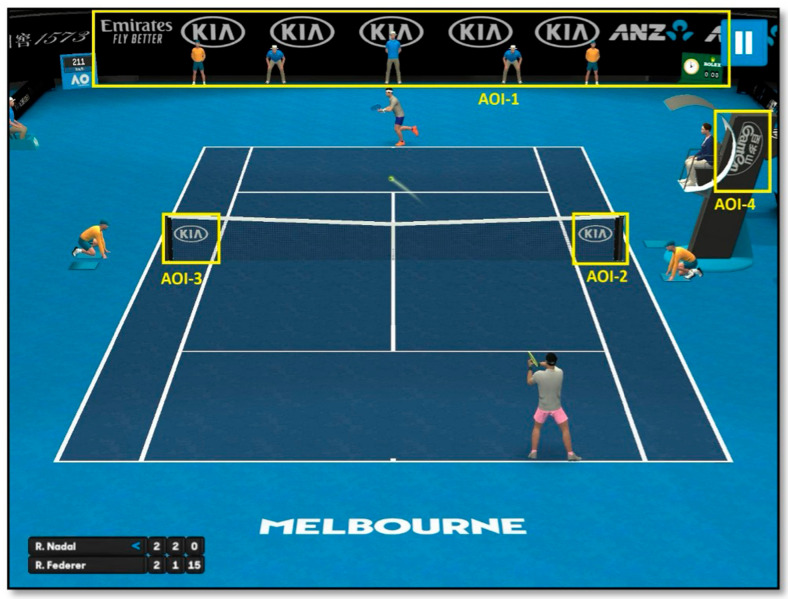
Areas of Interest (AOIs) defined in the mobile sports game.

The experimental stimuli consisted of four static, non-animated brand advertisements integrated into a free mobile tennis game available in the App Store. All ads appeared during active gameplay rather than in loading or menu screens, ensuring ecological validity and natural exposure. The advertisements were standardized in size (960 × 120 px), contrast, and duration of on-screen visibility (~10 s per rally) to maintain uniform visual salience across brands. AOIs (Areas of Interest) were defined according to the fixed screen layout of the game (see Figure 1), and all brand logos remained visible throughout gameplay, without overlapping the player interface or obstructing key game elements.

The selected brands (Kia, Emirates, Rolex, ANZ, Ganten) represent real sponsors commonly associated with international tennis tournaments. They were chosen based on their recognizability in global sports sponsorship contexts rather than local familiarity. To minimize excessive cognitive load during gameplay, a simple and easy-to-play tennis game was chosen. Logos and brands appearing on loading or menu screens were excluded from the analysis, focusing instead on in-game advertisements displayed during active play.

Four Areas of Interest (AOIs) were defined (see Figure 1):

- **AOI-1:** Banner advertisements positioned at the top of the court (e.g., Emirates).
- **AOI-2:** Side panels near the baseline (e.g., Kia).
- **AOI-3:** Side panels closer to the players (e.g., Kia).
- **AOI-4:** Rotating advertisement panel on the right side of the court (e.g., ANZ).

For each AOI, the following eye-tracking metrics were analyzed:

- **Time to First Fixation (TTFF):** Time (ms) until the first fixation on the AOI.
- **First Fixation Duration (FFD):** Duration (ms) of the first fixation on the AOI.
- **Total Fixation Duration (TFD):** Cumulative fixation duration (ms) on the AOI.
- **Total Fixation Count (TFC):** Number of fixations on the AOI.

These AOI-based analyses enabled a detailed examination of how visual attention was distributed across different in-game advertisements during active play.

### 2.4. Experimental Procedure

Data were collected using Tobii Pro Glasses 2 eye-tracking glasses and analyzed in Tobii Pro Lab software (**Pro Lab version 1.145**) [48,49]. Additionally, a 12-item questionnaire was administered. Prior to the session, participants were informed of the general purpose of the study, but the specific focus on in-app advertisements was not disclosed to avoid bias and preserve ecological validity.

**Figure 2:**
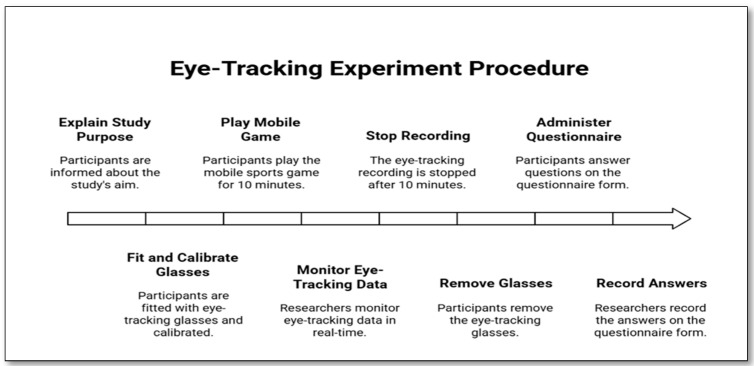
Timeline of the experimental procedure for tracking user experience in mobile games.

The experimental flow is summarized in Figure 2:

- **0 min:** Participants were welcomed, informed consent was obtained, and instructions were provided.
- **0–2 min:** Tobii Pro Glasses 2 were fitted and a three-point calibration was performed by having participants fixate on a wall marker until confirmation appeared in Tobii Pro Lab.
- **2–10 min:** Participants played the mobile sports game for ten minutes while their eye movements were recorded. Researchers monitored the data live on the connected computer.
- **10 min:** Recording was stopped, and participants removed the eye-tracking glasses.
- **10–20 min:** Participants completed the questionnaire assessing demographic variables (gender, MSGE, tennis interest) and brand recall (unaided and aided).

Each session lasted approximately 20 min per participant. The experimental design ensured internal validity by maintaining a consistent game sequence and identical advertisement placement across all participants. The duration of each session (approximately ten minutes) was intentionally limited to minimize fatigue and learning effects. In-game advertisements appeared at fixed spatial locations (AOIs) and remained static throughout gameplay, preventing order or exposure bias. Participants played the same level and difficulty setting, ensuring equal task demands and visual exposure for all. This controlled setup enhanced the reliability and comparability of visual attention and recall data across individuals.

### 2.5. Data Analysis

Demographic variables (gender, MSGE, and interest in tennis) were analyzed in IBM SPSS Statistics v.22. Gender was coded as male = 1, female = 2; MSGE = 1 (yes), 0 (no); tennis interest = 1 (yes), 0 (no). Prior to the experiment, participants were not informed about the brands featured in the game, ensuring that recall responses reflected spontaneous recognition rather than prior knowledge. Unaided recall was assessed by asking participants to freely list any brands they remembered from the game. Verbal-aided recall involved providing brand names orally and asking whether each was seen during gameplay, while visual-aided recall was conducted by showing brand logos and asking participants to confirm recognition. Responses were coded dichotomously (1 = recalled, 0 = not recalled) based on accurate identification of brands actually present in the game.

#### 2.5.1. Assumptions and Diagnostics

Prior to hypothesis testing, data normality was examined using the Kolmogorov–Smirnov test and skewness/kurtosis thresholds (±2, ±7) [50]. As the fixation metrics violated normality assumptions, preliminary group comparisons were conducted using non-parametric Mann–Whitney U tests, and effect sizes were computed as r = Z/√N following Rosenthal’s formulation [51]. Given the large number of comparisons, these non-parametric tests were treated as exploratory and interpreted without multiplicity correction. To rigorously test predictive relationships while accounting for the non-normal, nested structure of the data (multiple brands per participant), the primary confirmatory analysis was advanced to Generalized Linear Mixed Models (GLMM).

#### 2.5.2. Generalized Linear Mixed Models (GLMM)

To account for the nested structure of the data and to directly test the predictive relationship between visual attention and brand recall (RQ2), a Generalized Linear Mixed Model (GLMM) with a binomial distribution and logit link function was implemented. *Visual-Aided Recall* (0/1) served as the dependent variable. Visual-Aided Recall was selected as the primary outcome measure because it provides the most sensitive assessment of perceptual memory traces formed during high-velocity gameplay, minimizing retrieval failures often associated with purely verbal recall tasks.

Eye-tracking metrics (Total Fixation Duration and Time to First Fixation) were included as continuous predictors, while Brand (reflecting spatial placement), Gender, and Interest in Tennis were modeled as fixed effects. Participant ID was specified as a random intercept to account for repeated AOI-level observations within individuals. This approach effectively controls for within-subject correlations and reduces the Type-I error inflation that may arise from multiple independent comparisons. All mixed-effects analyses were conducted in R (version 4.3.×) using the lme4 package. Logistic GLMMs (glmer) were used for recall outcomes.

#### 2.5.3. Eye-Tracking Data Processing

Eye-tracking data were recorded at a sampling rate of 100 Hz using Tobii Pro Glasses 2 and processed in Tobii Pro Lab [49]. A three-point calibration procedure was employed, achieving an average accuracy below 0.5°. Recordings with more than 10% tracking loss were excluded from the analysis. Data preprocessing included automatic removal of blinks and signal losses longer than 150 ms. Fixations were identified using Tobii’s I-VT (Velocity-Threshold Identification) algorithm with a velocity threshold of 30°/s, a minimum fixation duration of 60 ms, and the default Tobii Pro Lab noise-reduction smoothing settings (gap fill < 75 ms).

#### 2.5.4. Metric Calculation

The analyzed metrics included Time to First Fixation (TTFF), First Fixation Duration (FFD), Total Fixation Duration (TFD), and Total Fixation Count (TFC) for each Area of Interest (AOI). Since all AOIs remained visible throughout gameplay, Time to First Fixation (TTFF) was calculated relative to the onset of each rally. To ensure temporal precision, ‘rally onset’ was operationally defined as the first video frame indicating racket–ball contact during the service motion. TTFF values were computed for each individual rally event to capture attention resets associated with the start of gameplay. These rally-level values were then averaged per participant for each AOI (Mean aggregation) to derive a stable latency metric and minimize variance caused by differing rally durations. Spatially, AOIs were defined as non-overlapping regions; in rare cases of gaze-point uncertainty near boundaries, fixations were assigned based on the AOI covering the foveal center (>50%pixel coverage).

#### 2.5.5. Visualization Framework and Standardization

Visualization and analysis were performed using Tobii Pro Lab and customized analytical routines following established visualization methodologies [38,39,42,44,52,53,54]. These frameworks emphasize the integration of temporal and spatial gaze mapping to ensure reproducibility and interpretability in human–computer interaction studies. Accordingly, the present visualization workflow combined static representations (e.g., heatmaps) with temporal analyses (e.g., scanpaths and gaze plots) to illustrate both spatial attention density and sequential gaze transitions.

To enhance interpretability, additional visual analytics methods were employed to examine both the spatial and temporal characteristics of attention distribution during gameplay. This approach aligns with the visualization principles outlined by Blascheck et al. [44] and Jacob and Karn [39], which highlight that graphical representations of gaze behavior enable the identification of perceptual focus and attention-shift patterns often obscured in purely statistical data. Similarly, Raschke et al. [38] and Burch et al. [42] demonstrated that integrating heatmaps with sequential gaze visualizations strengthens interpretability and ecological validity in dynamic environments such as mobile gaming.

In line with these recommendations, the study adopted a dual-level visualization framework: (1) static attention-density maps to depict fixation concentration across in-game advertisements, and (2) sequential visualizations to trace temporal gaze behavior throughout gameplay. Through these combined techniques, the study captured both the quantitative (e.g., fixation metrics) and qualitative (e.g., gaze transition paths) dimensions of visual attention, providing a comprehensive visualization-based understanding of how users allocate and shift attention in realistic, dynamic gaming environments. To ensure reproducibility and comparability, visualization parameters were standardized. Heatmaps were generated using a standard Gaussian kernel (radius ~50 px) and a duration-based accumulation metric to represent fixation density. To isolate significant attentional clusters, the transparency threshold was set to eliminate areas receiving negligible fixation duration (<5% of max). For scanpaths, fixation circles were scaled relative to duration, with saccade connections smoothed to illustrate gaze trajectories.

#### 2.5.6. Power Analysis

An a priori power analysis was conducted using G*Power 3.1 to determine the adequacy of the sample size. Assuming a medium effect size (d = 0.5), α = 0.05, and desired power of 0.80 for two-tailed independent comparisons, the required total sample size was estimated at 64 participants (32 per group). The present sample of 79 participants therefore provided sufficient statistical power (1 − β = 0.87) to detect medium-sized effects.

## 3. Results

### 3.1. Unaided Brand Recall

Participants’ unaided recall performance was first examined. When asked whether they noticed any advertisements during gameplay, 21 participants (26.6%) reported seeing at least one advertisement, while 58 (73.4%) reported seeing none. Among all brands displayed within the mobile sports game, *Kia* was the most frequently recalled brand (*n* = 18), whereas *ANZ* was the least recalled (*n* = 1) (Table 1).

Mann–Whitney U tests were used to compare unaided recall performance across gender, MSGE, and tennis interest groups. A significant difference was observed only for Emirates based on tennis interest (U = 552.50, Z = −2.15, *p* = 0.032). Participants not interested in tennis recalled Emirates more frequently (Mean Rank = 43.39) than those interested (Mean Rank = 38.69). No significant differences were found for Kia, ANZ, Rolex, or Ganten (*p* > 0.05) (Table 2).

### 3.2. Verbal-Aided Brand Recall

Verbal-aided recall results showed that *Kia* was again the most recalled brand (*n* = 45), followed by *Rolex* (*n* = 19), *Emirates* (*n* = 15), *ANZ* (*n* = 12), and *Ganten* (*n* = 5) (Table 3).

Gender-based comparisons revealed a significant difference only for *Rolex* (U = 527.00, Z = −3.04, *p* = 0.002), with males (Mean Rank = 44.79) recalling it more often than females (Mean Rank = 32.97). No significant differences were found for other brands or variables (Table 4).

### 3.3. Visual-Aided Brand Recall

When recall was supported by visual cues, *Kia* achieved the highest recognition rate (*n* = 75), followed by *ANZ* (*n* = 36), *Emirates* (*n* = 32), *Rolex* (*n* = 30), and *Ganten* (*n* = 15) (Table 5).

Gender differences were found only for *Rolex* (U = 469.50, Z = −3.36, *p* = 0.001), again favoring male participants. Based on tennis interest, *Ganten* recall differed significantly (U = 476.00, Z = −2.43, *p* = 0.015), with non-tennis participants demonstrating higher recall (Mean Rank = 46.80) than tennis-interested participants (Mean Rank = 37.35) (Table 6).

### 3.4. Eye-Tracking Metrics by AOIs

Participants’ eye movement data were analyzed across the four defined AOIs. Time to first fixation (TTFF), first fixation duration (FFD), total fixation duration (TFD), and total fixation count (TFC) were compared across gender, MSGE, and tennis interest variables.

As seen in Table 7, for AOI-1, significant differences were observed in total fixation duration (U = 413, Z = −2.34, *p* = 0.019) and total fixation count (U = 381.5, Z = −2.69, *p* = 0.007), with non-tennis participants showing longer and more frequent fixations (Table 7).

As seen in Table 8, for AOI-2, first fixation duration significantly differed by tennis interest (U = 431.5, Z = −2.14, *p* = 0.032), indicating that tennis-interested participants spent more time on initial fixations (Table 8).

As seen in Table 9, for AOI−3, significant differences emerged for TTFF in both MSGE (U = 387, Z = −2.30, *p* = 0.022) and tennis interest (U = 426, Z = −2.21, *p* = 0.027), with non-tennis and non-MSGE participants showing delayed first fixations (Table 9).

As seen in Table 10, for AOI-4, gender-based differences were found in TTFF (U = 526, Z = −2.36, *p* = 0.018) and FFD (U = 564.5, Z = −1.98, *p* = 0.048), with females showing longer first fixations and longer latency to fixate compared to males.

### 3.5. Visualization of Eye-Tracking Data

**Figure 3:**
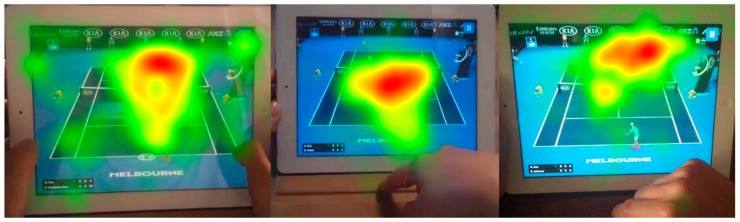
Heatmaps illustrating aggregated gaze density for brands located in AOI-2 (side-panel placement). Kernel density was generated using a 50-px Gaussian radius with Tobii I-VT fixation classification (30°/s). The intense central hotspot visually reflects the strong placement-driven effect confirmed by the GLMM results (Table 11).

To visualize the spatial distribution of visual attention during gameplay, fixation heatmaps were generated for representative participants. Warmer colors (red/yellow) indicate higher fixation frequency and duration, while cooler colors (green) represent lower levels of gaze concentration. As illustrated in Figure 3, participants’ gaze density primarily clustered in the central gameplay region and around the side panel area (AOI-2), where the KIA logo was displayed most prominently. These patterns confirm that visual attention naturally gravitated toward regions combining gameplay relevance and high ad visibility.

Consistent with prior visualization research showing that heatmaps effectively capture attentional density in complex interactive scenes [55,56], the present data reveal concentrated attention clusters in the top-banner and central gameplay zones. This supports the notion that ad visibility and perceptual salience jointly determine memory outcomes during gameplay.

**Figure 4:**
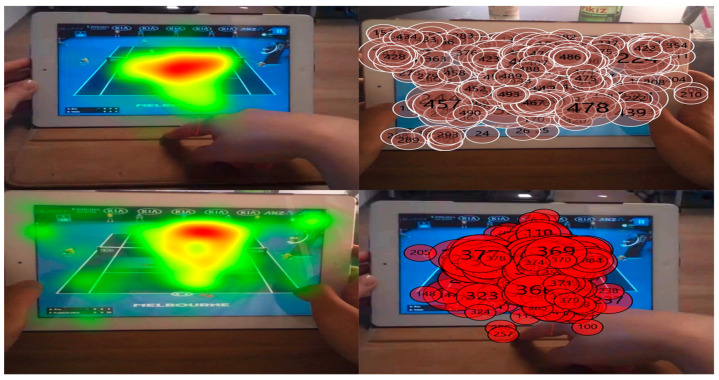
Gaze plots visualizing sequential attention allocation (saccade connections smoothed for clarity). Data represent typical scanpaths observed during active rallies. The scanpaths reveal that while participants often made early saccades to peripheral ads (AOI-1), these did not result in sustained dwell times. This visual pattern aligns with the statistical finding that ‘Time to First Fixation’ (TTFF) was not a significant predictor of recall (*p* > 0.05), whereas sustained engagement on task-relevant areas was crucial.

In addition to the fixation heatmaps, gaze plots were incorporated to provide a more detailed visualization of sequential gaze behavior and attention dispersion during gameplay. As shown in Figure 4, participants’ fixations followed consistent spatial and temporal patterns—initially focusing on the central gameplay area before shifting toward the top-banner advertising region (AOI-2), where the KIA logo was displayed. These combined visualizations illustrate how attention dynamically transitioned between gameplay and advertising elements, indicating that ads positioned in perceptually salient yet task-relevant zones can naturally capture attention without disrupting gameplay flow.

The scanpath sequences show how attention first stabilizes in gameplay-critical foveal regions before shifting toward top-banner ads. Such temporal trajectories illustrate how attention evolves dynamically—a finding aligned with visualization frameworks identifying gaze sequence data as key to decoding cognitive load and perceptual prioritization [44,57].

**Figure 5:**
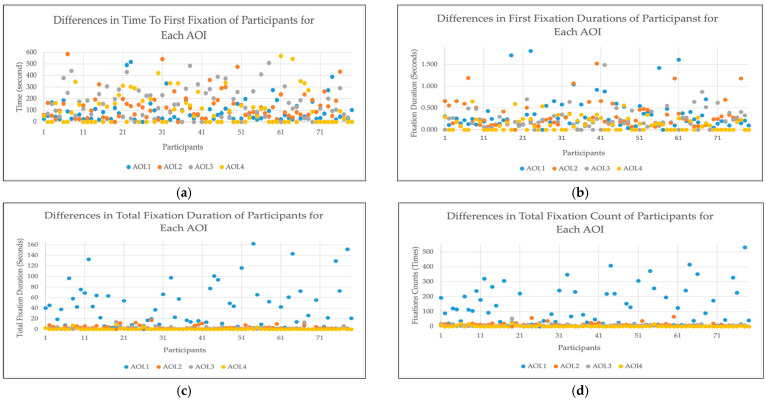
Scatter plots representing individual differences in eye-tracking metrics across Areas of Interest (AOIs): (**a**) Time to First Fixation (TTFF), (**b**) First Fixation Duration (FFD), (**c**) Total Fixation Duration (TFD), and (**d**) Total Fixation Count (TFC). Each dot represents a participant’s measurement for each AOI. Higher TTFF values indicate delayed attention allocation, whereas higher FFD, TFD, and TFC values reflect deeper visual engagement and repeated attention shifts within the corresponding regions.

Figure 5 presents the distribution of individual fixation metrics across the four Areas of Interest (AOIs). Higher Time to First Fixation (TTFF) values indicate that participants detected these regions later during gameplay, suggesting delayed attention allocation. The First Fixation Duration (FFD) data show how long participants maintained their initial focus on each AOI; longer durations imply more detailed visual inspection or cognitive processing. The Total Fixation Duration (TFD) values represent the cumulative time participants spent fixating on each AOI—higher values correspond to more sustained visual engagement. Finally, Total Fixation Count (TFC) indicates how frequently participants revisited a particular AOI, reflecting repeated attentional shifts or interest in that region. Zero values in any metric denote cases where no fixations occurred on the respective AOI. Collectively, these patterns illustrate how both perceptual salience and task relevance shape the dynamics of attention allocation during active gameplay, supporting the notion that advertising elements embedded within meaningful visual contexts are more likely to sustain and recapture gaze engagement.

### 3.6. Prediction of Brand Recall by Eye-Tracking Metrics

A Generalized Linear Mixed Models (GLMM) analysis was conducted to determine the predictors of brand recall. The results (Table 11) revealed that Brand Identity/Placement was the most significant predictor of recall (*p* < 0.001). Specifically, the Kia brand (integrated into the court) had significantly higher odds of being recalled compared to the reference brand (Emirates, top banner) (*B* = 3.11, *p* < 0.001), while Ganten showed significantly lower recall odds (*B* = −1.26, *p* = 0.003).

After controlling for brand placement, Total Fixation Duration (TFD) did not show a significant independent effect on recall (*p* = 0.672). This suggests that the *location* and *integration* of the advertisement (e.g., central vs. peripheral) are more critical for memory formation than marginal variations in fixation duration. Neither Gender nor Interest in Tennis showed a significant main effect in the multivariate model.

## 4. Discussion

This study examined the relationship between in-app advertising, brand recall, and visual attention in mobile sports games using eye-tracking technology. The findings provide novel insights into how demographic and involvement factors influence advertising effectiveness in interactive digital environments.

Firstly, the KIA brand achieved the highest recall across all three conditions—unaided, verbally aided, and visually aided—while ANZ had the lowest unaided recall scores. This outcome aligns with previous findings emphasizing the role of visual salience, spatial positioning, and repetition frequency in memory formation [35,36,58]. Advertisements located in high-visibility or foveal regions attract longer and more sustained fixations, which are often associated with stronger memory traces and higher recall [59]. These results confirm that effective in-game advertising depends not only on exposure frequency but also on the perceptual congruence between an ad’s visual salience and its spatial placement, allowing brands to integrate naturally into players’ attention streams during active gameplay. Visualization-based findings in similar studies further show that fixation density patterns are strong predictors of brand recognition and recall accuracy [46,56].

Secondly, demographic and behavioral variables significantly influenced recall performance. Male participants recalled Rolex more frequently than females, consistent with prior advertising and eye-tracking research indicating gender-based differences in visual attention and information processing [60,61,62]. Men tend to exhibit a more focal and form-oriented visual strategy, whereas women process information more holistically. In interpreting these findings, the potential influence of pre-existing brand familiarity must be explicitly acknowledged. Since globally recognized brands (e.g., Rolex, Kia) were used without collecting baseline familiarity ratings or employing foil brands (distractors) in the recall task, we cannot rule out that high aided recall scores partially reflect prior knowledge rather than exclusive in-game encoding. Without signal detection metrics (d’), it is possible that the reported recall rates are slightly inflated by a ‘response bias’ toward familiar logos. Thus, the recall values observed, particularly for luxury brands like Rolex, should be viewed as a combination of in-game attention and prior brand schema.

Interestingly, participants not interested in tennis recalled Emirates and Ganten more often than those with higher tennis interest. This inverse relationship can be explained through the Limited Capacity Model of Attention [63] and the Elaboration Likelihood Model [64]. Tennis-involved participants likely allocated more cognitive resources to task-related gameplay, reducing peripheral attention to ads, whereas less-involved participants directed more attention toward secondary visual stimuli. Visualization-driven analyses similarly demonstrate that task relevance and domain expertise shape gaze dispersion and scanning sequences across areas of interest [44,47].

Thirdly, the eye-tracking data revealed meaningful differences in attention allocation across the four Areas of Interest (AOIs). Participants with lower tennis involvement showed longer and more frequent fixations on AOI-1 (upper banner ads), while those with higher involvement displayed longer first fixation durations on AOI-2 (baseline panels). These results correspond with theories emphasizing that domain expertise and task relevance guide selective visual attention [65,66]. Moreover, fixation duration and timing are directly associated with cognitive load and information processing depth [67,68], suggesting that user involvement modulates attention during interactive gameplay.

It is important to acknowledge that because brands were fixed to specific AOIs (e.g., Kia was always located on the central net), the observed recall differences reflect a confounding of brand identity and spatial placement. Our GLMM analysis indicates that the strong performance of Kia is likely driven by its prime positioning within the player’s foveal field rather than intrinsic brand characteristics alone. Consequently, the results should be interpreted as placement-contingent: advertisements embedded in task-central gameplay zones (AOI-2/3) garner significantly higher recall than peripheral banner ads (AOI-1), regardless of the specific brand displayed.

Crucially, our GLMM analysis extends the descriptive findings by highlighting the dominance of ad placement over raw fixation duration. While descriptive statistics showed variations in fixation times, the multivariate model demonstrated that fixation duration alone does not guarantee recall (*p* > 0.05) once the brand’s position is controlled. This implies that high-visibility zones (like the central court area used by Kia) generate strong memory traces not merely because they are looked at longer, but because they are cognitively integrated into the gameplay task. This supports the ‘Task-Relevance’ hypothesis, suggesting that attention directed at task-relevant areas (AOI-2/3) is processed more deeply than attention on peripheral banners (AOI-1), even if fixation durations are comparable.

Beyond statistical comparisons, visualization-based analyses—including heatmaps, scanpaths, and gaze plots—added interpretive depth by revealing how attention dynamically shifted throughout gameplay. As discussed by Raschke et al. (2021) [38] and Kurzhals et al. (2019) [40], visualization-driven methods bridge the gap between numerical data and perceptual interpretation, allowing researchers to trace not only where participants looked but also how their attention evolved over time. Likewise, Blascheck et al. (2017) [44] emphasized that visual analytics can uncover micro-patterns of gaze concentration and transition that are often obscured in aggregate statistics. Integrating visual and quantitative analyses therefore enhances explanatory power and aligns this study with contemporary visualization frameworks in eye movement research.

From a visualization standpoint, the convergence between high-density heatmap clusters and scanpath trajectories clarifies how cognitive attention oscillates between gameplay and advertising stimuli. The correspondence between these clusters and the strong recall performance for KIA supports the argument that visual attention patterns can predict recognition and memory outcomes [56]. These findings are consistent with broader visual analytics literature emphasizing temporal–spatial mapping and attention tracking as essential for interpretability in human–computer interaction studies [52,53,69,70].

Collectively, the visualization results highlight how each analytical layer contributes to understanding the cognitive–perceptual mechanisms behind brand recall. The heatmap analyses (Figure 3) illustrate spatial clustering of gaze density around ad-rich regions, while the gaze plot and scanpath sequences (Figure 4) reveal the temporal evolution of attention between gameplay and advertisements. Taken together, these visualization-driven findings strengthen the connection between perceptual salience, cognitive processing, and brand recall, underscoring the interpretive value of combining spatial and temporal visual analytics in interactive media research.

Theoretically, this research extends brand recall studies from passive viewing contexts to active and interactive digital environments. While most prior studies relied on laboratory-based passive observation, this study improved ecological validity by collecting data during real gameplay [34,65,66]. The results demonstrate that recall mechanisms in interactive media differ fundamentally from those in passive contexts, where attention is externally guided. From a visualization perspective, the findings reinforce the emerging view that eye movement visualization bridges cognitive attention models with perceptual behavior in dynamic environments [45,57]. From a specific HCI and vision research perspective, these findings challenge the traditional ‘fixation-memory’ linearity often assumed in passive media. Our GLMM results demonstrate that in interactive environments, task-relevance overrides visual salience. The fact that fixation duration (TFD) lost its predictive power once placement was controlled suggests that in active vision, memory encoding is not a function of ‘dwell time’ but of ‘task-integration.’ This advances current eye movement models by showing that high-cognitive-load tasks decouple the standard link between foveal attention duration and recall, a critical insight for designing attention-aware interfaces.

Practically, the findings highlight that in-game advertising effectiveness depends on the interplay between ad placement, visual salience, and audience characteristics. Ads placed in high-visibility areas produced higher recall, and demographic as well as involvement factors significantly moderated advertising impact. Consequently, marketers should optimize not only spatial placement but also cognitive alignment with their target audiences’ interests and motivational profiles. Visualization-based analysis can further support such optimization by identifying perceptual blind spots and attention bottlenecks within mobile interfaces [71]. Implementing such strategies may enhance both brand awareness and long-term brand loyalty [22,33].

## 5. Conclusions

This study provides clear evidence that brand recall in mobile sports games is shaped by visual salience, ad placement, and user engagement. The KIA brand achieved the highest recall across all conditions, confirming that ads positioned in high-visibility zones generate stronger memory traces. In contrast, ANZ showed the weakest unaided recall, underscoring the importance of perceptual distinctiveness.

Critically, our multivariate analysis (GLMM) established that ad placement is a more robust predictor of recall than fixation duration alone. This suggests that in active gaming contexts, task-integration (e.g., placing ads in the central gameplay zone) is more vital for memory encoding than mere dwell time. Eye-tracking results further revealed that gender and sport involvement influence how attention is distributed during active gameplay: male participants recalled Rolex more frequently, and less-involved players paid greater attention to peripheral brands such as Emirates and Ganten. These findings indicate that attention allocation in interactive media is not random but guided by cognitive focus and motivational relevance.

Overall, the study demonstrates that effective in-game advertising depends on aligning visual design, spatial positioning, and audience characteristics, offering both theoretical and practical insights into how brands can capture and sustain attention in dynamic digital environments. Importantly, these findings establish a conceptual bridge between visual attention models and real-world gaming behavior, emphasizing how perceptual and motivational processes interact within interactive media contexts.

By integrating visual analytics into eye-tracking research, future studies can further connect cognitive mechanisms of attention with perceptual visualization, broadening the methodological scope of gaze-based advertising research. Combining quantitative fixation data with visual tools such as heatmaps and scanpaths provides a methodological framework linking where participants look with what they remember. This integrated approach supports future research employing interactive and attention-aware visualization systems to capture the dynamic relationship between perception, cognition, and advertising effectiveness [45,57,71].

## 6. Limitations and Future Research

Although this study provides novel insights into brand recall and attention mechanisms in mobile sports games, several limitations should be acknowledged. This study employed a single sports game and a relatively short exposure duration, which may have limited its generalizability to other genres or longer gameplay sessions. Moreover, the sample consisted mainly of young adults recruited through convenience sampling, reducing representativeness for the broader gaming population. While data were collected in a controlled setting to ensure measurement precision, this laboratory-like environment may not fully capture the ecological dynamics of natural gaming contexts. Future research could replicate this design across multiple game types and more diverse participant groups, ideally incorporating field experiments to enhance ecological validity and generalizability. Because brands were not counterbalanced across AOI locations, the observed recall differences may reflect placement and frequency effects. Future studies should rotate brand positions or equalize exposure across AOIs to isolate true brand-level effects.

This study also examined a limited number of in-app advertisements; therefore, the results may vary across different game genres, advertising formats, or levels of task difficulty. Eye-tracking measures capture visual attention but not the full depth of cognitive or emotional processing. Future studies could integrate multimodal physiological measures such as EEG, galvanic skin response, or heart rate to better understand the affective and neural correlates of in-game attention. Additionally, cross-cultural and longitudinal designs could reveal how cultural context, media literacy, and repeated exposure influence advertising effectiveness over time.

Moreover, aided recall measures in this study included only brands that actually appeared in the game, without the use of lure or foil brands. This design choice may have inflated aided recall accuracy by not accounting for false recognitions or brand familiarity effects. Future studies should incorporate non-presented brands in aided recall tasks to estimate false-alarm rates and compute more precise recognition metrics, such as signal detection indices (d′, c). Such an approach would provide a more nuanced understanding of genuine brand recognition versus guessing tendencies.

Finally, future research should consider developing adaptive advertising models that dynamically adjust visual salience and placement based on real-time user engagement. Furthermore, researchers are encouraged to employ interactive or linked-view visualization systems that enable dynamic analyses of gaze and recall over time [58]. Such systems would enhance both ecological validity and analytical depth in complex, real-world gaming environments.

## Figures and Tables

**Table 1:** Unaided recall of brands.

	Frequency		Percentage	
Brands	Remembered	Not Remembered	Remembered	Not Remembered
Kia	18	61	22.8	77.2
Emirates	4	75	5.1	94.6
ANZ	1	78	1.3	98.7
Rolex	9	70	11.4	88.6
Ganten	2	77	2.5	97.5
*n*	79		100	

**Table 2:** Mann–Whitney U results of unaided recall of brands.

**Gender**	**Kia**	**Emirates**	**ANZ**	**Rolex**	**Ganten**
U	585.0	630.5	718.0	725.5	527.0
Z	−1.244	−1.683	−0.825	−1.179	−1.174
*p*	0.214	0.092	0.409	0.238	0.240
**MSGE**	**Kia**	**Emirates**	**ANZ**	**Rolex**	**Ganten**
U	568.0	589.5	580.0	561.5	570.0
Z	−0.341	−0.015	−0.582	−0.584	−0.829
*p*	0.733	0.988	0.560	0.559	0.407
**Interest in Tennis**	**Kia**	**Emirates**	**ANZ**	**Rolex**	**Ganten**
U	626.5	552.5	598.5	607.5	609.5
Z	−0.008	−2.14	−1.61	−0.38	−0.70
*p*	0.994	0.032 *	0.107	0.698	0.482

* *p* < 0.05.

**Table 3:** Verbal-aided recall of brands.

	Frequency		Percentage	
Brands	Remembered	Not Remembered	Remembered	Not Remembered
Kia	45	34	43	57
Emirates	15	64	19	81
ANZ	12	67	15.2	84.8
Rolex	19	60	24.1	75.9
Ganten	5	74	6.3	93.7
*n*	79		100	

**Table 4:** Mann–Whitney U results of verbal-aided recall of brands.

**Gender**	**Kia**	**Emirates**	**ANZ**	**Rolex**	**Ganten**
U	585.0	630.1	718.0	527.0	751.0
Z	−1.945	−1.786	−0.546	−3.035	−0.024
*p*	0.052	0.074	0.585	0.002 *	0.981
**MSGE**	**Kia**	**Emirates**	**ANZ**	**Rolex**	**Ganten**
U	495.5	582.0	549,0	582,5	579.5
Z	−1.242	−0.133	−0.744	−0.114	−0.281
*p*	0.214	0.894	0.457	0.909	0.779
**Interest in Tennis**	**Kia**	**Emirates**	**ANZ**	**Rolex**	**Ganten**
U	606.0	620.0	613.5	599.0	603.0
Z	−0.268	−0.113	−0.237	−0.414	−0.622
*p*	0.789	0.910	0.812	0.679	0.534

* *p* < 0.05.

**Table 5:** Visual-aided recall of brands.

	Frequency		Percentage	
Brands	Remembered	Not Remembered	Remembered	Not Remembered
Kia	75	4	93.7	6.3
Emirates	32	47	40.5	59.5
ANZ	36	43	45.6	54.4
Rolex	30	49	38	62
Ganten	15	60	19	81
*n*	79		100	

**Table 6:** Mann–Whitney U results of visual-aided recall of brands.

**Gender**	**Kia**	**Emirates**	**ANZ**	**Rolex**	**Ganten**
U	711.5	674.5	735.5	469.5	676.0
Z	−0.959	−0.910	−0.191	−3.356	−1.117
*p*	0.338	0.363	0.849	0.001 *	0.264
**MSGE**	**Kia**	**Emirates**	**ANZ**	**Rolex**	**Ganten**
U	579.5	475.5	555.0	527.0	503.0
Z	−0.281	−1.518	−0.457	−0.845	−1.444
*p*	0.779	0.129	0.647	0.398	0.149
**Interest in Tennis**	**Kia**	**Emirates**	**ANZ**	**Rolex**	**Ganten**
U	611.5	591.0	588.5	534.0	476.0
Z	−0.402	−0.463	−0.488	−1.210	−2.431
*p*	0.688	0.643	0.625	0.226	0.015 *

* *p* < 0.05.

**Table 7:** Mann–Whitney U results of eye movements on AOI-1.

**Gender**	**Time to 1st fixation**	**1st fixation duration**	**Total fixation duration**	**Total fixation count**
U	697.0	687.0	750.5	671.0
Z	−0.549	−0.649	−0.015	−0.809
*p*	0.583	0.516	0.988	0.418
**MSGE**	**Time to 1st fixation**	**1st fixation duration**	**Total fixation duration**	**Total fixation count**
U	571.0	586.5	506.5	530.5
Z	−0.214	−0.039	−0.941	−0.671
*p*	0.129	0.647	0.398	0.149
**Interest in Tennis**	**Time to 1st fixation**	**1st fixation duration**	**Total fixation duration**	**Total fixation count**
U	589.0	595.5	413.0	381.5
Z	−0.416	−0.345	−2.341	−2.685
*p*	0.678	0.730	0.019 *	0.007 *

* *p* < 0.05.

**Table 8:** Mann–Whitney U results of eye movements on AOI-2.

**Gender**	**Time to 1st fixation**	**1st fixation duration**	**Total fixation duration**	**Total fixation count**
U	743.0	685.5	626.5	650.5
Z	−0.090	−0.665	−1.254	−1.016
*p*	0.928	0.506	0.210	0.310
**MSGE**	**Time to 1st fixation**	**1st fixation duration**	**Total fixation duration**	**Total fixation count**
U	461.0	583.0	565.5	578.0
Z	−1.455	−0.079	−0.276	−0.136
*p*	0.146	0.937	0.782	0.892
**Interest in Tennis**	**Time to 1st fixation**	**1st fixation duration**	**Total fixation duration**	**Total fixation count**
U	605.0	431.5	625.5	545.5
Z	−0.241	−2.140	−0.016	−0.893
*p*	0.810	0.032 *	0.987	0.372

* *p* < 0.05.

**Table 9:** Mann–Whitney U results of eye movements on AOI-3.

**Gender**	**Time to 1st fixation**	**1st fixation duration**	**Total fixation duration**	**Total fixation count**
U	725.0	738.0	657.0	645.0
Z	−0.271	−0.141	−0.952	−1.075
*p*	0.787	0.888	0.341	0.282
**MSGE**	**Time to 1st fixation**	**1st fixation duration**	**Total fixation duration**	**Total fixation count**
U	387.0	530.5	548.5	583.0
Z	−2.297	−0.676	−0.470	−0.079
*p*	0.022 *	0.499	0.639	0.937
**Interest in Tennis**	**Time to 1st fixation**	**1st fixation duration**	**Total fixation duration**	**Total fixation count**
U	426.0	511.5	560.0	519.0
Z	−2.206	−1.272	−0.735	−1.189
*p*	0.027 *	0.203	0.462	0.235

* *p* < 0.05.

**Table 10:** Mann–Whitney U results of eye movements on AOI-4.

**Gender**	**Time to 1st fixation**	**1st fixation duration**	**Total fixation duration**	**Total fixation count**
U	526.0	564.5	598.0	654.0
Z	−2.362	−1.977	−1.624	−1.037
*p*	0.018 *	0.048 *	0.104	0.300
**MSGE**	**Time to 1st fixation**	**1st fixation duration**	**Total fixation duration**	**Total fixation count**
U	533.0	526.0	489.5	524.5
Z	−0.673	−0.762	−1.196	−0.783
*p*	0.501	0.446	0.232	0.434
**Interest in Tennis**	**Time to 1st fixation**	**1st fixation duration**	**Total fixation duration**	**Total fixation count**
U	577.0	555.5	551.5	536.0
Z	−0.572	−0.826	−0.872	−1.055
*p*	0.567	0.409	0.383	0.292

* *p* < 0.05.

**Table 11:** GLMM Analysis Results Predicting Visual-Aided Brand Recall.

Predictor	*B* (Coef.)	Std. Err.	z	*p*	Odds Ratio
(Intercept)	−0.051	0.590	−0.09	0.932	0.95
Brand: Kia (vs. Emirates)	3.109	0.541	5.74	<0.001	22.4
Brand: Ganten (vs. Emirates)	−1.255	0.416	−3.01	0.003	0.28
Total Fixation Duration (TFD)	−0.003	0.006	−0.42	0.672	0.99
Time to First Fixation (TTFF)	0.002	0.002	0.97	0.334	1.00
Gender	−0.812	0.461	−1.76	0.078	0.44
Interest in Tennis	0.774	0.432	1.79	0.073	2.16

## Data Availability

The de-identified gaze data and analysis code will be deposited on OSF/Zenodo upon acceptance.
